# Nedosiran in primary hyperoxaluria subtype 3: results from a phase I, single-dose study (PHYOX4)

**DOI:** 10.1007/s00240-023-01453-3

**Published:** 2023-04-28

**Authors:** David S. Goldfarb, John C. Lieske, Jaap Groothoff, Gesa Schalk, Kerry Russell, Shuli Yu, Blaz Vrhnjak

**Affiliations:** 1 New York Harbor Department of Veterans Affairs Medical Center, New York University School of Medicine, New York, NY USA; 2 Mayo Clinic, Rochester, MN USA; 3 Academic Medical Center (AMC), Amsterdam, The Netherlands; 4 Kindernierenzentrum, Bonn, Germany; 5 Dicerna Pharmaceuticals, Inc., a Novo Nordisk Company, Lexington, MA USA; 6 Novo Nordisk, Søborg, Denmark

**Keywords:** Chronic kidney impairment, Nephrolithiasis, Kidney calculi, Hyperoxaluria, RNAi, Gene expression, Nephrology, Urology

## Abstract

Nedosiran is an *N*-acetyl-D-galactosamine (GalNAc)–conjugated RNA interference agent targeting hepatic lactate dehydrogenase (encoded by the *LDHA* gene), the putative enzyme mediating the final step of oxalate production in all three genetic subtypes of primary hyperoxaluria (PH). This phase I study assessed the safety, pharmacokinetics (PK), and pharmacodynamics (PD) of subcutaneous nedosiran in patients with PH subtype 3 (PH3) and an estimated glomerular filtration rate  ≥ 30 mL/min/1.73 m^2^. Single-dose nedosiran 3 mg/kg or placebo was administered in a randomized (2:1), double-blinded manner. Safety/tolerability, 24-h urinary oxalate (Uox) concentrations, and plasma nedosiran concentrations were assessed. The main PD endpoint was the proportion of participants achieving a > 30% decrease from baseline in 24-h Uox at two consecutive visits. Six participants enrolled in and completed the study (nedosiran, n = 4; placebo, n = 2). Nedosiran was well-tolerated and lacked safety concerns. Although the PD response was not met, 24-h Uox excretion declined 24.5% in the nedosiran group and increased 10.5% in the placebo group at Day 85. Three of four nedosiran recipients had a > 30% reduction in 24-h Uox excretion during at least one visit, and one attained near‐normal (i.e., ≥ 0.46 to < 0.60 mmol/24 h; ≥ 1.0 to < 1.3 × upper limit of the normal reference range) 24-h Uox excretion from Day 29 to Day 85. Nedosiran displayed predictable plasma PK. The acceptable safety and trend toward Uox-lowering after single-dose nedosiran treatment enables further clinical development of nedosiran in patients with PH3 who currently have no viable therapeutic options. A plain language summary is available in the supplementary information.

**Supplementary Information:**

The online version contains supplementary material available at 10.1007/s00240-023-01453-3.

## Introduction

Primary hyperoxaluria (PH) is a family of three rare autosomal recessive genetic disorders–PH1, PH2, PH3–each characterized by a specific deficiency in hepatic glyoxylate metabolism [1, 2]. Chronic, elevated levels of endogenous oxalate in plasma and urine are the hallmark of all three PH subtypes, which is associated with damage to the kidneys and other organs [2]. Current evidence suggests PH may affect approximately 1 in 38,600 people in the United States (carrier frequency 1:58), with PH3 having an estimated prevalence of 1 in 135,866 people and a carrier frequency of 1:185 [3]. PH3 accounts for approximately 10% of observed PH cases, although underdiagnosis and/or incomplete penetrance may underestimate PH3 prevalence [2–4].

Previously, PH3 patients have been described as having relatively stable renal function over time, but recent findings from the European Hyperoxaluria Consortium (OxalEurope) Registry and Rare Kidney Stone Consortium Primary Hyperoxaluria (RKSC PH) Registry suggest that this is not always the case [4, 5]. More than half of PH3 cases (50–89%) present with renal stones before age 5 years [5, 6], 14–29% develop chronic kidney impairment [4, 5, 7], and 3–4% develop kidney failure by age 40 years [3, 5]. It is not unusual for patients with PH3 to have symptomatic stone disease throughout their life [4, 5]. Multiple occurrences of painful kidney stones requiring urologic intervention represent one of the most troubling aspect of PH for both patients and their caregivers [8–10]. Currently, PH3 is managed based solely on supportive options [11].

Belostotsky et al. identified mutations in *DHDPSL*, the gene encoding mitochondrial 4-hydroxy-2-oxoglutarate aldolase (HOGA1), as responsible for PH3 [12–14]. This enzyme catalyzes the final step in the metabolic pathway of hydroxyproline to pyruvate; however, loss of function mutations result in glyoxylate (and thus oxalate) overproduction [12].

Nedosiran is an RNA interference (RNAi) agent that inhibits hepatic lactate dehydrogenase (LDH) expression (encoded by the *LDHA* gene) [15]. Evidence suggests this enzyme may mediate the final common step in oxalate production for all three forms of PH [16, 17]. In clinical trials, nedosiran administration resulted in a marked reduction in 24-h urinary oxalate (Uox) and plasma oxalate (Pox) in patients with PH1 and demonstrated acceptable tolerability [15, 18].

The primary objective of this phase I study (PHYOX4) was to evaluate the safety and tolerability of single-dose nedosiran in patients with PH3.

## Methods

### Study design and conduct

PHYOX4 was a multinational, randomized, double‐blind, placebo‐controlled trial assessing the safety, pharmacokinetics (PK), and pharmacodynamics (PD) of a single dose of subcutaneous nedosiran in participants with PH3 (ClinicalTrials.gov number: NCT04555486). It was conducted between January 2021 and September 2021 in accordance with the provisions of the Declaration of Helsinki, Good Clinical Practice Guidelines of the International Conference on Harmonisation, and all applicable laws and regulations. Written informed consent was obtained from all adult participants and the parents or legal guardians of participating children. All children assented as appropriate. An independent Data and Safety Monitoring Committee (DSMC) monitored the trial for safety. Those who completed PHYOX4 were eligible for screening into the open-label extension study DCR-PHXC-301 to receive nedosiran on an ongoing basis (ClinicalTrials.gov Identifier: NCT04042402; PHYOX3), with DSMC approval.

### Study population

Key eligibility criteria for enrollment were a documented PH3 diagnosis confirmed by genotyping, age ≥ 6 years at time of informed consent, a history of ≥ 1-stone event within the last 12 months, 24-h Uox excretion ≥ 0.7 mmol (adjusted per 1.73 m^2^ body surface area [BSA] in participants age < 18 years) in two collections performed during the screening period, an estimated glomerular filtration rate (eGFR) ≥ 30 mL/min/1.73 m^2^ BSA, and a willingness to use contraception if applicable. Stone events were defined as a renal stone requiring medical intervention (e.g., outpatient procedures such as lithotripsy or hospitalization or inpatient surgical intervention for confirmed stone-related pain and/or complications), stone passage with/without hematuria, or renal colic requiring medication. An eGFR at screening ≥ 30 mL/min/1.73 m^2^ was calculated using the Chronic Kidney Disease Epidemiology Collaboration (CKD-EPI) formula [19] in participants age ≥ 18 years or using the multivariate formula by Schwartz in participants age 6–17 years [20]. Less than 20% variation between two 24-h urinary creatinine measurements in the screening period was required. Individuals who did not achieve < 20% variation between the two screening values could were permitted a second round of urine collection to meet this requirement. If they did not meet this requirement after two collections, they were excluded from participation.

Key exclusion criteria included prior or scheduled kidney or liver transplantation, dialysis, or any condition/comorbidity that would affect the study or patient safety. Individuals with a Pox level > 30 μmol/L, liver function abnormalities, hepatitis-/HIV-positive, and/or anti-dsDNA-positive were ineligible. We also excluded individuals who had used an RNAi drug within the last 6 months, a history of reactions to oligonucleotide-based therapy (or hypersensitivity to nedosiran), or received an investigational drug in a clinical study within 4 months (or five times the drug’s half-life) of PHYOX4.

### Randomization and treatment

Drug allocation was undertaken centrally according to the drug randomization number/scheme generated by the sponsor using SAS software (SAS Institute Inc., Cary, NC, USA), which assigned participants to nedosiran and placebo groups in a 2:1 ratio. Implementation of the randomization scheme was achieved using an Interactive Web Response System. Each participant received a single dose of study intervention on Day 1. A nedosiran dose of 3 mg/kg for all PH3 participants ≥ 12 years of age was used to allow comparisons between results of this study and results from a study of patients with PH1 and PH2 (PHYOX1, NCT03392896) [15]. Participants 6–11 years of age were given a 3.5 mg/kg dose, based on PK/PK-PD simulations (total dose did not exceed 136 mg). The placebo was 0.9% saline for injection administered at a volume to match that of the active intervention.

Participants avoided vitamin C supplements (and multivitamins) for 24 h before and during urine collections, as well as oxalate-rich foods and strenuous exercise for 24 h before each blood draw [21]. All participants followed standard of care for PH (e.g., hyperhydration, oral potassium citrate intake).

### Assessments and endpoints

The primary objective of this study was to assess the safety and tolerability of nedosiran in patients with PH3. Safety was assessed via adverse event (AE) reporting, physical examinations, electrocardiograms, vital signs, and clinical laboratory tests conducted at screening and throughout the study. AEs were coded using the Medical Dictionary for Regulatory Activities (version 23.1 or higher). Key AE variables were the incidence and severity of any treatment-emergent AEs (until end of study; Day 85), serious AEs (until 30 days after last day of participation), and AEs of special interest (AESIs). Injection site reactions (ISRs) were considered AESIs and defined as signs or symptoms at the injection site, with a time to onset ≥ 4 h post-dose. ISRs were evaluated according to the Common Terminology Criteria for Adverse Events version 5.0.

Prespecified secondary objectives were Uox excretion and nedosiran PK characterization. Nedosiran PD was assessed by measuring changes in 24-h Uox excretion at baseline (screening) and at Days 29, 43, 57, and 85 post-dose. The predefined PD responder definition was the proportion of participants achieving a > 30% decrease from baseline in 24-h Uox on two consecutive visits. To maintain the integrity of all analyses related to 24-h Uox measurement, on-treatment 24-h urinary creatinine excretion values were required to be within 20% of baseline, defined as the mean of the two screening values; collections that did not meet this criterion were repeated. Collections with a reported duration of < 22 h or > 26 h at screening or on treatment were considered invalid, and participants were asked to repeat them.

We also explored 24-h Uox/creatinine ratio (calculated from 24-h urine samples); kidney stone events (12 months prior to screening and during study); levels of urinary creatinine, citrate, calcium, phosphate, and magnesium; and fluid intake (4- to 7-day period before each 24-h urine collection).

Serial blood samples were collected pre-dose and at prespecified post-dose times for plasma nedosiran concentration measurements. Individual blood concentration–time data were used to calculate nedosiran PK parameters using non-compartmental analysis. The primary PK parameters were maximum measured plasma concentration (C_max_), time to C_max_ (T_max_), time of last measurable concentration (T_last_), and areas under the concentration–time curve (AUC) over 24 h from time of dosing (AUC_0-24_) and from time of dosing to the last measurable blood concentration (AUC_0-last_).

Antibodies to nedosiran and anti–double-stranded DNA antibodies were measured at screening, at Days 1 and 85 post-dose, and at the visit for any participants prematurely discontinuing from the study.

### Statistics

Since the primary endpoints were safety and tolerability, no formal sample size estimations were performed. A sample size of six participants (four in the nedosiran arm and two in the placebo arm) was considered sufficient to provide an initial assessment of the safety and PK/PD profile of nedosiran in patients with PH3. Analyses of safety, stone events, and fluid intake were evaluated in all participants exposed to study intervention, analyzed according to the intervention received. Urine analyses were evaluated in the modified intent-to-treat population (mITT), defined as all participants who were randomly assigned, received a partial or full dose of study intervention, and had post-dose 24-h Uox values collected at ≥ 2 consecutive visits. Pharmacokinetic analysis was assessed in all participants who received a full dose of nedosiran and had ≥ 1 evaluable post-dose PK assessment.

Three prespecified sensitivity analyses were performed for the primary PD endpoint using BSA-adjusted 24-h Uox excretion for all participants, all Uox excretion concentrations (including those not meeting completeness criteria), and last observation carried forward (LOCF) imputation for missing 24-h Uox excretion data if they were missing or if no measurement met the completeness criteria at a scheduled visit (N.B., if the Day 29 sample was missing or incomplete, baseline was not carried forward).

## Results

### Study population

Twelve individuals were screened for participation in the study (Fig. S1). Six of these individuals were considered screen failures, primarily because of low 24-h Uox excretion (< 0.7 mmol/1.73 m^2^ BSA). Six participants were randomized into the study (nedosiran, n = 4; placebo, n = 2), all of whom were included in the safety and the mITT populations. Four of six eligible participants entered the open-label extension study PHYOX3.

Five of six participants (83.3%) were white and age > 18 years (Table 1). Four of six participants were males, including both participants in the placebo group. Mean (± SD) age overall was 42.5 (± 19.8) years and was slightly higher in the nedosiran group than the placebo group (44.8 [13.6] vs 38.0 [36.8] years). Baseline weight and BSA were comparable between groups. All four participants in the nedosiran group were age ≥ 12 years (the minimum age was 25 years) and thus received the 3 mg/kg dosage.

**Table 1:** Baseline demographic and clinical characteristics (safety population)

Status	Nedosiran (N = 4)	Placebo (N = 2)	Total (N = 6)
Age, mean (SD), years	44.8 (13.6)	38.0 (36.8)	42.5 (19.8)
Age category, n (%)			
< 12 years	0	0	0
< 18 years	0	1 (50)	1 (17)
≥ 18 years	4 (100)	1 (50)	5 (83)
Male, n (%)	2 (50)	2 (100)	4 (67)
Race, n (%)			
White	3 (75)	2 (100)	5 (83)
Not reported	1 (25)	0	1 (17)
Weight, mean (SD), kg	77.13 (17.65)	71.00 (12.73)	75.08 (15.14)
Body surface area, mean (SD), m^2^	1.87 (0.23)	1.80 (0.17)	1.85 (0.20)
24-h Uox excretion, mean (SD), mmol/day^a^	1.30 (0.60)	1.02 (0.08)	1.21 (0.49)
eGFR, mean (SD), mL/min/1.73 m^2 b^	89 (34)	76 (1)	85 (28)
eGFR ≥ 60 mL/min/1.73 m^2 b^, n (%)	3 (75)	2 (100)	5 (83)
CKD stage, n (%)			
Stage 1	2 (50)	2 (100)	4 (67)
Stage 2	1 (25)	0	1 (17)
Stage 3A	1 (25)	0	1 (17)
≥ 1 kidney stone event, n (%)	4 (100)	2 (100)	6 (100)
No. kidney stone events in last 12 months, mean (SD)	1.0 (0)	1.0 (0)	1.0 (0)
Renal and urinary disorders, n (%)	4 (100)	2 (100)	6 (100)
Nephrolithiasis	2 (50)	1 (50)	3 (50)
Hematuria	1 (25)	1 (50)	2 (33)
Renal colic	1 (25)	1 (50)	2 (33)
Urinary calculus	1 (25)	0	1 (17)
Time since PH diagnosis, mean (SD), months	83.3 (124.4)	42.5 (61.3)	69.7 (102.3)
≥ 1 prior procedure related to nephrocalcinosis and/or nephrolithiasis, n (%)	3 (75)	2 (100)	5 (83)
Surgical and medical procedures	3 (75)	2 (100)	5 (83)
Lithotripsy	2 (50)	2 (100)	4 (67)
Renal stone removal	2 (50)	1 (50.0)	3 (50)
Ureteral stent insertion	0	1 (50.0)	1 (17)
Ureteral stent removal	1 (25)	0	1 (17)
Ureteric calculus removal	1 (25)	0	1 (17)
Urinary calculus removal	0	1 (50)	1 (17)
Investigations	1 (25)	1 (50)	2 (33)
Ureteroscopy	1 (25)	1 (50)	2 (33)
Pox, mean (SD), mg/L	0.43 (0.09)	0.50 (0.06)	0.45 (0.08)

*BSA* body surface area, *CKD* chronic kidney disease, *CKD-EPI* chronic kidney disease epidemiology collaboration, *eGFR* estimated glomerular filtration rate, *No.* number, *PH* primary hyperoxaluria, *Pox* plasma oxalate, *SD* standard deviation, *Uox* urinary oxalate

^a^BSA-adjusted Uox was used for participants < 18 years of age

^b^For calculation of eGFR, CKD-EPI equation was used for participants ≥ 18 years of age, and 2012 Schwartz Equation was used for participants < 18 years of age

Mean 24-h Uox excretion was 1.21 mmol at baseline and was similar between the nedosiran and placebo groups (1.30 mmol and 1.02 mmol, respectively). All participants had baseline eGFR ≥ 45 mL/min/1.73 m^2^. Four of six participants (66.7%) had CKD Stage 1, and, in the nedosiran group, one participant had CKD Stage 2, and one participant had CKD Stage 3A. All participants had experienced one kidney stone event in the last year and had associated renal/urinary disorders (stone, renal colic, or hematuria). Pox levels were within the normal reference range in all participants. Moreover, the time since diagnosis was approximately twice as long in the nedosiran as the placebo group (83.3 [124.4] months vs 42.5 [61.3] months). The most frequent prior/concomitant medications were urologic drugs, such as those for stone prevention. And the most common prior procedures or investigations were lithotripsy, renal stone removal, and ureteroscopy in both groups.

### Safety

Four participants were exposed to a single dose of nedosiran, and two participants were exposed to a single dose of placebo. The mean (± SD) dose of nedosiran was 230.8 (± 54.76) mg.

Three of four participants (75%) in the nedosiran group and two participants in the placebo group had ≥ 1 treatment-emergent AE (TEAE; Table 2). None were treatment-related, serious, fatal, or led to study withdrawal. All TEAEs were mild in nature, and no AESIs (injection site reactions) occurred.

**Table 2:** TEAEs in participants with PH subtype 3 who received single-dose nedosiran or placebo (safety population)

SOC and preferred term, n (%)	Nedosiran (N = 4)	Placebo (N = 2)
Any TEAE	3 (75)	2 (100)
Musculoskeletal/connective tissue disorders	2 (50)	0
Back pain	2 (50)	0
Skin/subcutaneous tissue disorders	2 (50)	0
Contact dermatitis	1 (25)	0
Scar pain	1 (25)	0
General disorders and administration site conditions	1 (25)	0
Pyrexia	1 (25)	0
Renal/urinary disorders	1 (25)	1 (50)
Urinary calculus	1 (25)	0
Nephrolithiasis	0	1 (50)
Renal pain	0	1 (50)
Infections and infestations	0	1 (50)
Nasopharyngitis	0	1 (50)

*AE* adverse event, *PH* primary hyperoxaluria, *SOC* system organ class, *TEAE* treatment-emergent AE

There were no treatment-related trends in vital signs for nedosiran or placebo, and no clinically relevant changes in hematology, clinical chemistry, coagulation studies, or urinalysis were observed. Back pain was the only TEAE in > 1 participant (two in nedosiran group). These TEAEs involved one instance of back tenderness in the right kidney region (in absence of other abnormal physical exam findings) and one instance of lower back pain, neither of which was considered related to nedosiran. Both instances were mild and resolved. One participant who received nedosiran had a high QTcF interval (452 ms) on electrocardiogram at Day 85. As their screening electrocardiogram showed a high QTcF (455 ms) and other measurements during the study were normal, this TEAE was not considered related to nedosiran. No other abnormal ECG findings were detected.

No antidrug antibodies were detected in any of the plasma samples collected from nedosiran-treated participants.

### Pharmacodynamics 

In the primary analysis, no participants in the mITT had a > 30% reduction from baseline in mean 24-h Uox excretion over two consecutive visits. In sensitivity analyses, one participant met the efficacy endpoint of > 30% reduction in mean 24-h Uox excretion over two consecutive visits when BSA-adjusted Uox was used for all participants. No participants met the endpoint when completeness criteria or LOCF imputation methods were used.

**Fig. 1:**
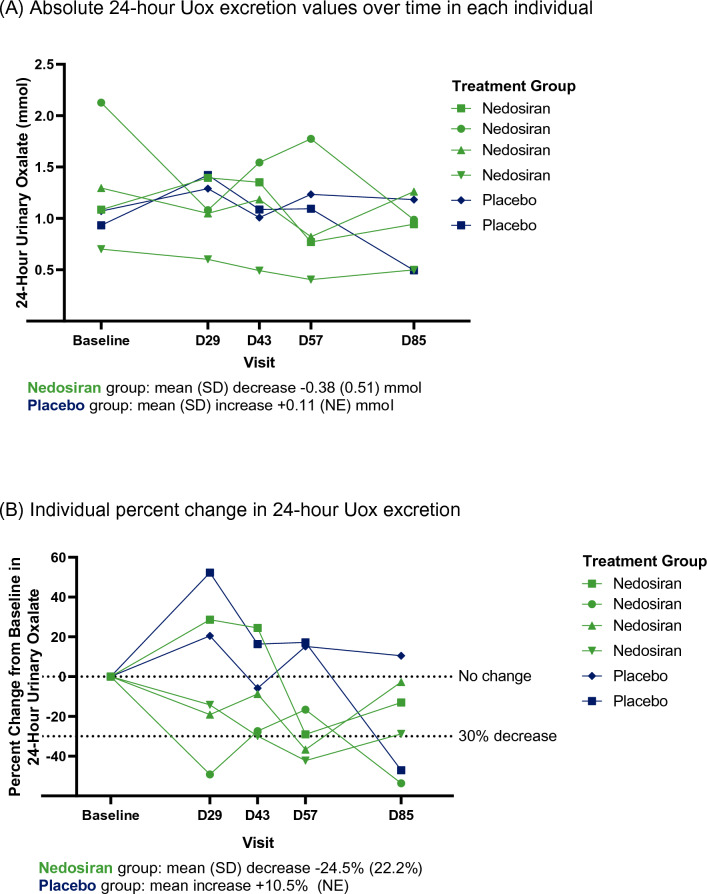
Individual changes in 24-h Uox excretion from baseline to Day 85 after single-dose administration of nedosiran or placebo (mITT population). *D* Day, *mITT* modified intention-to-treat, *NE* not estimable, *SD* standard deviation, *Uox* urinary oxalate. **A** Absolute 24-h Uox excretion values over time in each individual, **B** Individual percent change in 24-h Uox excretion

Figure 1 shows absolute and percent change from baseline in 24-h Uox excretion values for each participant over time. All four participants on nedosiran had an absolute reduction in 24-h Uox excretion (mean [± SD] reduction, −0.38 [± 0.51] mmol; median [range] reduction,  −0.17 [−1.14 to −0.03] mmol) from baseline to Day 85; their mean (± SD) percentage reduction in 24-h Uox excretion at Day 85 was 24.5% (± 22.2). Three participants in the nedosiran group had > 30% decreases in 24-h Uox at a single visit. These reductions in 24-h Uox excretion occurred between Days 29 and 85. One participant had BSA-adjusted near-normal Uox excretion (< 0.6 mmol/24 h) on Days 43 and 85 and normal Uox excretion on Day 57 (< 0.46 mmol/24 h). In contrast, Uox excretion increased by 0.11 mmol/24 h (or 10.5%) in the placebo group.

Mean (± SD) change from baseline to Day 85 in 24-h urinary oxalate-to-creatinine ratios were −28.5 (± 27.4) mmol/mol in the nedosiran group and 11.5 mmol/mol in the placebo group (Fig. S2). There were no clinically relevant changes from baseline to Day 85 in individual concentrations of 24-h urinary citrate, calcium, phosphate, and magnesium (Fig. S3).

One participant (25%) in the nedosiran group and one participant (50%) in the placebo group had kidney stone events during the study. There was one stone event in the nedosiran group and three stone events in the placebo group, of which two were concurrent (i.e., occurring in the same 4-week period).

### Pharmacokinetics

The arithmetic mean (± SD) plasma nedosiran concentration–time profile on a semi-log scale and the comparison of nedosiran AUC_0-last_ in participants with PH3 and PH1/PH2 are shown in Figs. S4 and S5, respectively. Following single 3 mg/kg subcutaneous dose administration, nedosiran was absorbed into the systemic circulation, attaining a median maximal plasma concentration approximately 8 h post-dose. Following single subcutaneous administration of nedosiran 3 mg/kg, PH3 participants from this clinical trial had a similar nedosiran AUC_0-last_ to PH1/PH2 participants of the previous PHYOX1 trial [15] (Fig. S5).

## Discussion

Recent epidemiologic data show that PH3 prevalence and burden have been underestimated [3–5, 8, 9, 22]. The identification of additional mutations in *DHDPSL* [13, 14], the *HOGA1* gene, and the lack of molecular screening across the entire *DHDPSL* coding region among the idiopathic stone-forming population suggests that PH3 is probably more widespread than anticipated [3]. All of our PHYOX4 participants had experienced recent kidney stone events, the cardinal sign of PH3 [4, 5]. Kidney function was generally preserved, although one participant had Stage 2 chronic kidney impairment and one participant had Stage 3A chronic kidney disease. In the first years after identification of the causative gene in 2010, PH3 patients had initially presented with symptoms in early childhood and had subsequently had mild reductions in eGFR relative to patients with PH1 and PH2 [3, 5]. However, there have been rare instances of kidney failure associated with PH3, possibly exacerbated by multiple stone removal surgeries which predispose to kidney damage [3, 22].

This phase I study is the first to examine targeted therapy with RNAi in patients with PH3. A single dose of nedosiran was safe and well-tolerated in this cohort, as evidenced by a lack of treatment-related TEAEs. The AE profile of nedosiran was consistent with previously reported clinical data on nedosiran [15, 18], with the exception of ISRs, which were *not* detected in PHYOX4. Only mild TEAEs were observed, and no safety risks were identified. At the single 3-mg/kg nedosiran dosage, the plasma nedosiran PK exposure in our PH3 participants was consistent with that found in PH1/PH2 patients, suggesting that nedosiran absorption and disposition are not affected by the PH subtype [15].

Twenty-four–hour Uox excretion is the accepted biomarker of PH disease burden, and in PH1, a higher 24-h excretion is associated with a greater likelihood of kidney failure [23, 24]. The predetermined PD endpoint in PHYOX4 was not met because no participants maintained the > 30% reduction in 24-h Uox excretion over two consecutive visits. However, despite similar or a tendency toward higher 24-h Uox excretion in the nedosiran group than the placebo group at baseline, 24-h Uox excretion declined by 24.5% in the nedosiran group and increased by 10.5% in the placebo group at Day 85. Three participants in the nedosiran group had a > 30% reduction in 24-h Uox excretion from baseline at one visit. One participant achieved normal (< 0.46 mmol/24 h) 24-h Uox excretion during the study. A similar reduction in Uox was not observed in the two participants in the placebo group. Finally, participants only received a single nedosiran dose and repeated nedosiran administration over several months, which possibly resulted in more complete suppression of hepatic LDH expression and, hence, a reduction in Uox excretion.

Firm conclusions from PHYOX4 are limited by the small sample size, which was based on safety, not efficacy, evaluations. A single-dose study of such a short duration is inadequate to draw conclusions regarding the effect on new stone formation/events, which are ubiquitous in PH3 patients. In addition, although participants were asked to avoid oxalate-rich foods, the diet was not controlled and adherence to this restriction was not measured. Therefore, we cannot speculate whether dietary variation throughout the study might have influenced Uox excretion and affected the results, although we are not certain that dietary oxalate content is an important determinant of Uox excretion in patients with PH3. Despite these limitations, our preliminary data warrant further investigation regarding the potential benefits of multiple dose nedosiran for PH3 patients in PHYOX3.

In conclusion, this randomized, double‐blind, placebo‐controlled trial indicated that a single subcutaneous dose of nedosiran was safe and well-tolerated in patients with PH3, with predictable PK. The nedosiran PD data was consistent with its mechanism of action as an inhibitor of hepatic LDH predicted to reduce hepatic oxalate generation and, hence, 24-h Uox excretion. Emerging preliminary data on the effect of multi-dose nedosiran in patients with PH3 will be shared in a separate publication.

### Supplementary Information

Below is the link to the electronic supplementary material.

Supplementary file1 (DOCX 459 KB)

## Data Availability

Because of the sensitive nature of the data collected for this study, the data set will not be made available to other researchers.
