# Correction to: Nedosiran in primary hyperoxaluria subtype 3: results from a phase I, single-dose study (PHYOX4)

**DOI:** 10.1007/s00240-023-01455-1

**Published:** 2023-06-05

**Authors:** David S. Goldfarb, John C. Lieske, Jaap Groothoff, Gesa Schalk, Kerry Russell, Shuli Yu, Blaz Vrhnjak

**Affiliations:** 1 New York Harbor Department of Veterans Affairs Medical Center, New York University School of Medicine, New York, NY USA; 2 Mayo Clinic, Rochester, MN USA; 3 Academic Medical Center (AMC), Amsterdam, The Netherlands; 4 Kindernierenzentrum, Bonn, Germany; 5 Dicerna Pharmaceuticals, Inc., a Novo Nordisk Company, Lexington, MA USA; 6 Novo Nordisk, Søborg, Denmark

**Correction to: Urolithiasis (2023) 51:80** 10.1007/s00240-023-01453-3

The original version of this article unfortunately contained a mistake. The copyright holder for this article was incorrectly given as

© This is a U.S. Government work and not under copyright protection in the US; foreign copyright protection may apply

but should have been

© The Author(s) 2023

The original article has been corrected.

