# Supplementary material for: Nedosiran in primary hyperoxaluria subtype 3: results from a phase I, single-dose study (PHYOX4)
Source: Urolithiasis. 2023 Apr 28;51(1):80. doi: 10.1007/s00240-023-01453-3 (PMC10147791; doi:10.1007/s00240-023-01453-3)
Supplement: Supplementary file 1 — Supplementary file1 (DOCX 459 KB) [file 240_2023_1453_MOESM1_ESM.docx]

**Supplementary Appendix**

This appendix has been provided by the authors to give readers additional information about their work.

Supplement to: David S. Goldfarb, John Lieske, Jaap Groothoff, et al. Nedosiran in Primary Hyperoxaluria Subtype 3: Results from a Phase I, Single-dose Study (PHYOX4)

**Plain language summary**

Primary hyperoxaluria (PH) is a group of three rare genetic diseases called PH1, PH2 and PH3. People living with PH make too much of a natural chemical called oxalate, which can – in large enough quantities – form kidney stones. Too much oxalate can also damage their kidneys and other organs. Currently, there is no cure for PH3. Nedosiran is an investigational medicine which aims to stop kidney stone formation by reducing oxalate production. In people living with PH1, nedosiran was found to be safe and shown to reduce oxalate production.

To test nedosiran treatment for PH3, researchers recruited six participants living with PH3 to participate in a new clinical trial called PHYOX4. The participants were randomly divided into two groups and received one injection of either nedosiran (four participants) or a placebo (a liquid containing no medicine; two participants). The main aim of the trial was to detect any unsafe side-effects caused by nedosiran treatment. Another aim was to measure the amount of urine oxalate excreted in the patients’ urine at different intervals during the trial. A reduction of at least 30% in oxalate levels over two back-to-back visits was needed to show that nedosiran treatment was effective.

There were no treatment-related unsafe side-effects in any of the participants after nedosiran or placebo injection. The researchers concluded that nedosiran was well tolerated by all participants. None of the participants given nedosiran achieved the goal of 30% reduction in oxalate over two back-to-back visits. However, all four participants given nedosiran showed a reduction in oxalate levels over the 12-week measurement period (an average reduction of 24.5% was seen). Three of the participants given nedosiran showed a reduction of more than 30% in oxalate levels at least once during the trial.

Overall, this trial shows that nedosiran is safe and well tolerated in people living with PH3. It also showed that oxalate levels can decrease after one injection of nedosiran. As the study goal for lowering of oxalate levels on back-to-back visits was not met, more research is needed to learn if nedosiran can help people with PH3.


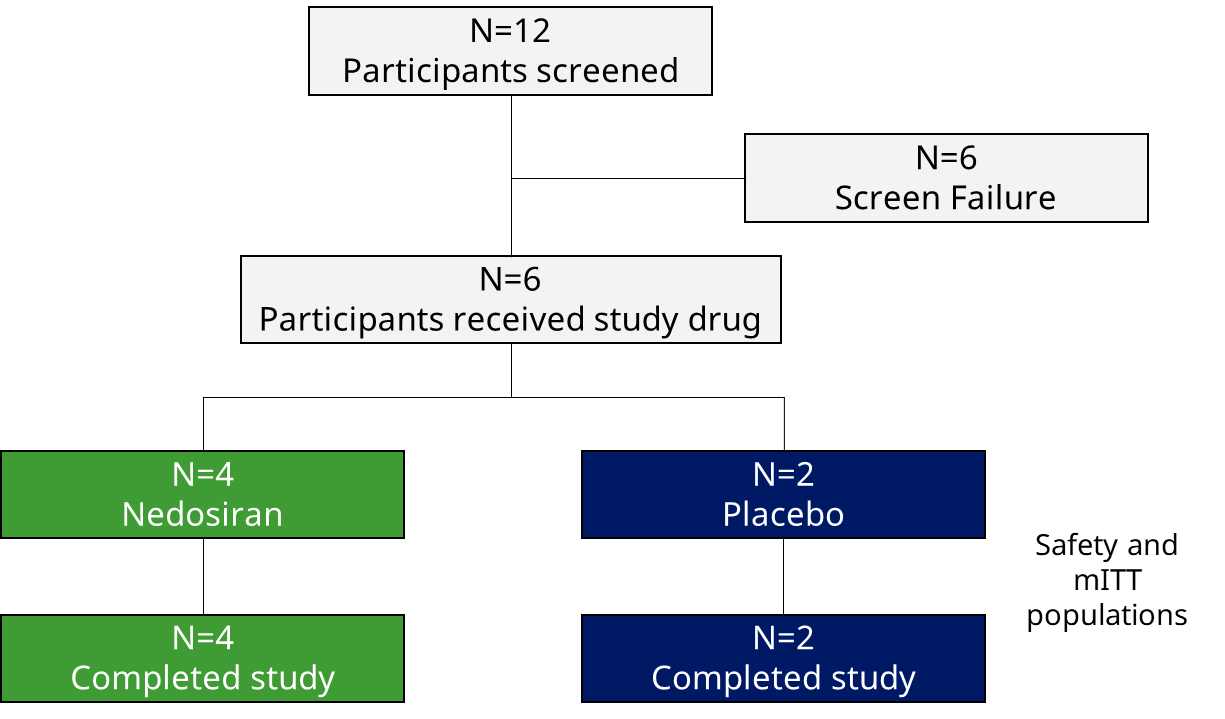


**Fig. S1** Flow of individuals through PHYOX4

*mITT* modified intention-to-treat


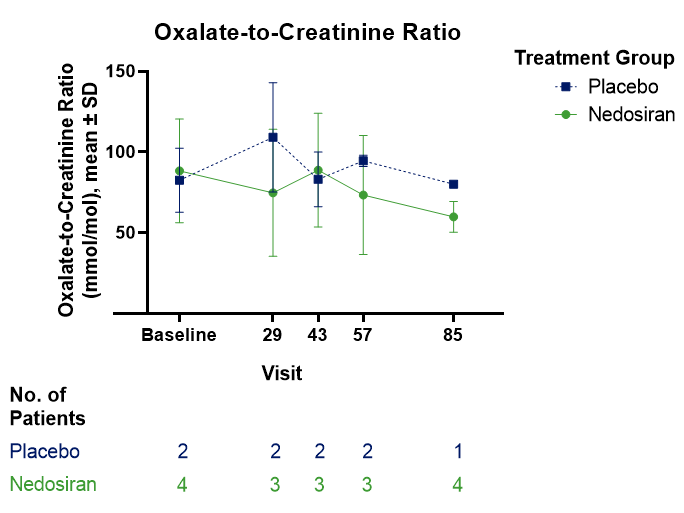


**Fig. S2** Mean (SD) oxalate-to-creatinine ratio over time (mITT population)

*mITT* modified intention-to-treat; *No.* number, *SD* standard deviation


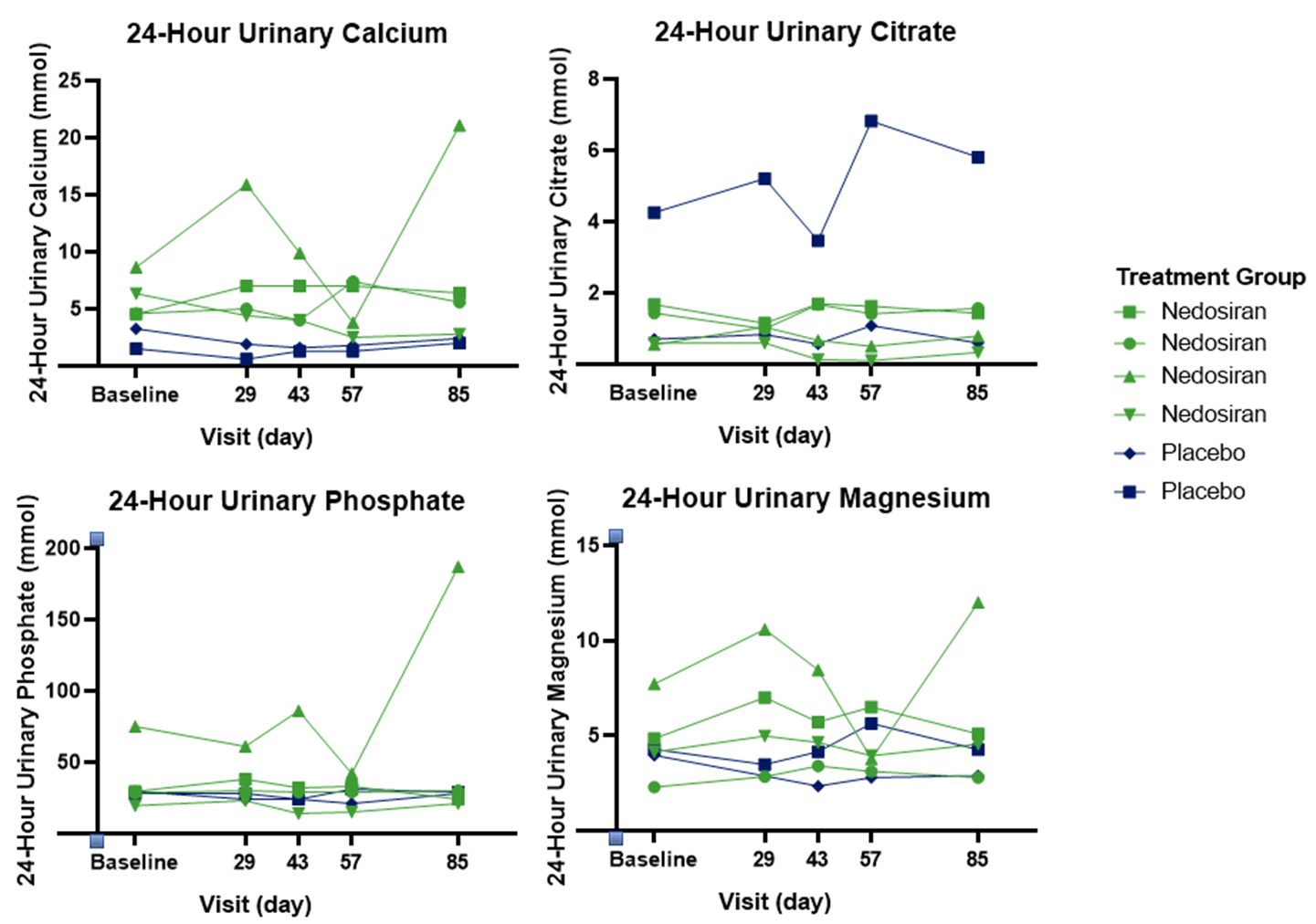


**Fig. S3** Individual 24-hour urinary citrate, calcium, phosphate, and magnesium (mITT population)

*mITT* modified intention-to-treat


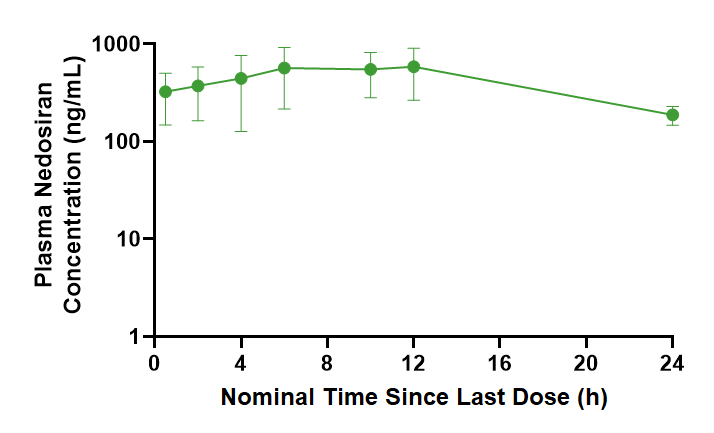


**Fig. S4** Semi-log plots of arithmetic mean (+SD) plasma concentrations of nedosiran following a single subcutaneous dose of 3 mg/kg nedosiran in participants with PH subtype 3

*PH* primary hyperoxaluria, *SD* standard deviation

Note: Dots and lines represent the mean profiles at nominal time points, error bars represent the standard deviation


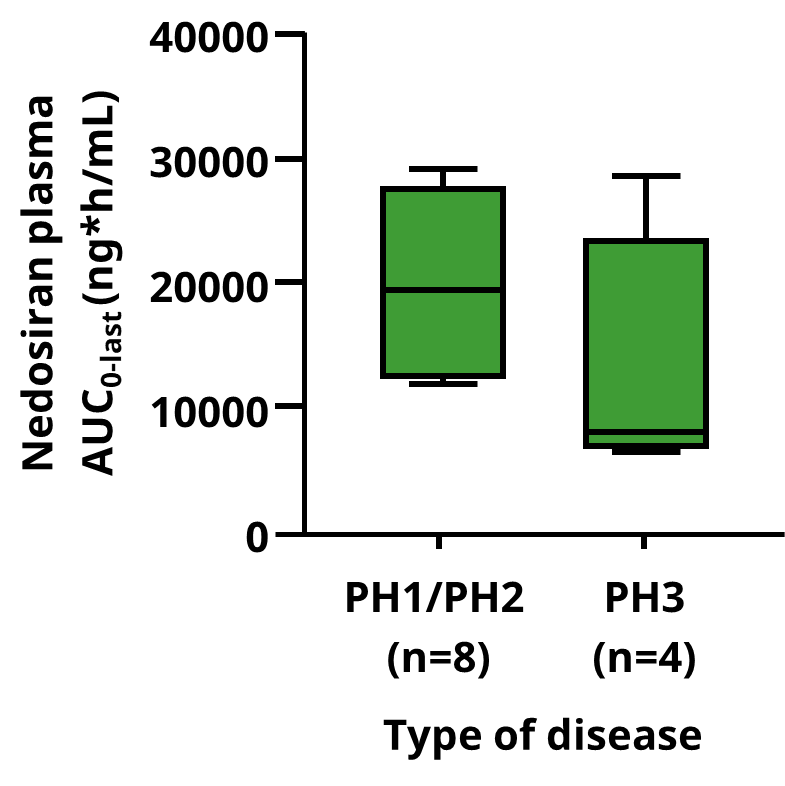


**Fig. S5** Comparison of plasma nedosiran AUC_0-last_ in participants with PH subtype 3 with patients with PH1/PH2 following a single subcutaneous dose of nedosiran 3 mg/kg

*AUC_0-last_* area under the concentration-time curve (AUC) from time of dosing to the last measurable blood concentration, *PH* primary hyperoxaluria

Note: The solid line inside the boxes represents the median of the data, hinges (top and bottom of the boxes) represent the 25^th^ and 75^th^ percentiles of the data, the top and bottom whiskers extend to the largest and smallest values that are within 1.5 times interquartile range

The nedosiran PK exposure data in PH1/PH2 patients were obtained in clinical trial PHYOX1
